# IL‐17A promotes lung fibrosis through impairing mitochondrial homeostasis in type II alveolar epithelial cells

**DOI:** 10.1111/jcmm.17600

**Published:** 2022-10-29

**Authors:** Huijuan Xiao, Liang Peng, Dingyuan Jiang, Yuan Liu, Lili Zhu, Zhen Li, Jing Geng, Bingbing Xie, Xiaoxi Huang, Jing Wang, Huaping Dai, Chen Wang

**Affiliations:** ^1^ Department of Pulmonary and Critical Care Medicine, Center of Respiratory Medicine, China‐Japan Friendship Hospital, School of Clinical Medicine Peking University Beijing China; ^2^ Department of Pulmonary and Critical Care Medicine, Center of Respiratory Medicine, China‐Japan Friendship Hospital; National Center for Respiratory Medicine; National Clinical Research Center for Respiratory Diseases, Institute of Respiratory Medicine, Chinese Academy of Medical Sciences Peking Union Medical College Beijing China; ^3^ Beijing Key Laboratory for Immune‐Mediated Inflammatory Diseases, Institute of Medical Science China‐Japan Friendship Hospital Beijing China; ^4^ Department of Respiratory and Critical Care Medicine Zhongnan Hospital of Wuhan University Wuhan China; ^5^ Medical Research Center Beijing Chaoyang Hospital Affiliated to Capital Medical University Beijing China; ^6^ State Key Laboratory of Medical Molecular Biology, Department of Physiology, Institute of Basic Medical Sciences Chinese Academy of Medical Sciences School of Basic Medicine Peking Union Medical College Beijing China

**Keywords:** IL‐17A, mitochondrial dysfunction, PTEN‐induced putative kinase 1, pulmonary fibrosis, type II alveolar epithelial cells

## Abstract

The dysfunction of type II alveolar epithelial cells (AECIIs), mainly manifested by apoptosis, has emerged as a major component of idiopathic pulmonary fibrosis (IPF) pathophysiology. A pivotal mechanism leading to AECIIs apoptosis is mitochondrial dysfunction. Recently, interleukin (IL)‐17A has been demonstrated to have a pro‐fibrotic role in IPF, though the mechanism is unclear. In this study, we report enhanced expression of IL‐17 receptor A (IL‐17RA) in AECIIs in lung samples of IPF patients, which may be related to the accumulation of mitochondria in AECIIs of IPF. Next, we investigated this relationship in bleomycin (BLM)‐induced PF murine model. We found that IL‐17A knockout (IL‐17A^−/−^) mice exhibited decreased apoptosis levels of AECIIs. This was possibly a result of the recovery of mitochondrial morphology from the improved mitochondrial dynamics of AECIIs, which eventually contributed to alleviating lung fibrosis. Analysis of in vitro data indicates that IL‐17A impairs mitochondrial function and mitochondrial dynamics of mouse primary AECIIs, further promoting apoptosis. PTEN‐induced putative kinase 1 (PINK1)/Parkin signal‐mediated mitophagy is an important aspect of mitochondria homeostasis maintenance. Our data demonstrate that IL‐17A inhibits mitophagy and promotes apoptosis of AECIIs by decreasing the expression levels of PINK1. We conclude that IL‐17A exerts its pro‐fibrotic effects by inducing mitochondrial dysfunction in AECIIs by disturbing mitochondrial dynamics and inhibiting PINK1‐mediated mitophagy, thereby leading to apoptosis of AECIIs.

## 1 INTRODUCTION

Pulmonary fibrosis (PF) is the end‐stage clinical manifestation of various chronic interstitial lung diseases with known or unknown causes and is pathologically characterized by remodelling of normal lung anatomy, deposition of extracellular matrix, and phenotypic changes of alveolar epithelial cells and fibroblasts.^1^ The most common and most serious form of PF is idiopathic PF (IPF), which is an unexplained progressive interstitial pneumonia. The median survival period for IPF patients without lung transplantation is about 3 years, and its morbidity and mortality are both increasing steadily.^2^ Because the unclear aetiology and pathogenesis make IPF difficult to treat or cure, there is an urgent need for new effective therapies to improve the prognosis of patients.^3^, ^4^

The pathogenesis of IPF is the result of multiple genetic and environmental risk factors that lead to repeated micro‐damages such as apoptosis of alveolar epithelial cells, especially the type II alveolar epithelial cells (AECIIs).^5^ These micro‐injuries initiate the abnormal communication between epithelial cells and fibroblasts, induction of matrix‐producing myofibroblasts, and considerable extracellular matrix accumulation with lung parenchymal tissue remodelling.^6^ Increasing research has suggested that the mitochondrial dysfunction caused by dysregulated mitochondrial dynamics and mitophagy is a key hallmark of AECIIs quality control in IPF pathogenesis.^7^ In IPF lungs, AECIIs showed a degree of dysmorphic and dysfunctional mitochondria that was associated with decreased expression levels of PTEN‐induced putative kinase 1 (PINK1), a ubiquitin kinase that regulates mitophagy.^8^ Moreover, increased levels of mitochondrial DNA (mtDNA) are detected in the bronchoalveolar lavage fluid and plasma of IPF patients.^9^ Recognizing and better understanding the mechanisms related to mitochondrial quality control in AECIIs could possibly lead to new therapeutic approaches for IPF.

Interleukin (IL)‐17A is the prominent cytokine of the IL‐17 family. It activates its downstream signalling pathway by binding to a heterodimer that is mainly formed by interleukin‐17A receptor A (IL‐17RA).^10^, ^11^ Numerous studies have indicated that IL‐17A contributes to the process of PF. In IPF tissues, IL‐17A expression was observed in areas that indicate disease activity, such as regenerating epithelium,^12^ and is also elevated in both the bronchoalveolar lavage of IPF patients and bleomycin (BLM)‐induced PF mouse model.^13^, ^14^ Mitochondrial dysfunction mediated by IL‐17A has been reported in several disease models, including rheumatoid arthritis (RA),^15^ vitiligo,^16^ and asthma.^17^ However, the effects of IL‐17A on mitochondrial function of AECIIs in IPF are not clear. In this study, we found that IL‐17A can impair the mitochondrial respiratory function of primary mouse AECIIs, possibly from disordered fusion/fission dynamics and diminished PINK1‐mediated mitophagy, which further increases apoptosis of AECIIs and thereby promotes PF.

## 2 MATERIALS AND METHODS

### 2.1 IPF tissues

Lung tissue samples were obtained through the Pulmonary Transplantation Department of China‐Japan Friendship Hospital. Samples were surgical remnants of lungs explanted from IPF patients (*n* = 8) who underwent pulmonary transplantation and donors (*n* = 5). Informed consent was obtained from all patients. This study was officially approved by the Ethics Committee of Medical School of China‐Japan Friendship Hospital (Licence No. 2019‐123‐K85‐1).

### 2.2 Animal treatments

Specific pathogen‐free male C57BL/6 mice (6–8 weeks old) were purchased (Vital River Laboratory Animal Technology Company, Beijing, China). IL‐17A knockout mice (IL‐17A^−/−^) of C57/BL6 background (6–8 weeks old) were contributed by Professor Huang of Beijing Chaoyang Hospital (Beijing, China). Genetic deletion of IL‐17A does not produce any endogenous lung phenotype and is not related to derangements of lung physiology, nor is it embryonically lethal in these mice. They produce litters of appropriate Mendelian size, are fertile and healthy, and present with no apparent morphology abnormalities. To establish the PF mouse model, mice were randomly assigned to either intratracheal administration of 1.5 mg/kg BLM (Nippon Kayaku Company, Tokyo, Japan) or equivalent volume (50 μl) of 0.9% saline as previously reported.^18^ Mice were sacrificed for lung harvest on day 21 after installation, the left lungs were used for staining and the right lungs were used for extracting protein or DNA. The immunohistochemical staining results of IL‐17A and IL‐17RA in lung tissues of mice in each group are shown in the Supplementary Materials (Figure S1B). All experimental procedures were approved by the Institutional Animal Care and Use Committee of the China‐Japan Friendship Hospital (Licence No. zryhyy21‐21‐06‐01).

### 2.3 Isolation of mice primary AECIIs, cell culture, and lentivirus transfection

Primary AECIIs were isolated from healthy male mice and mice in the animal experiments, as previously reported.^19^ Briefly, each mouse lung tissue was digested with 3 ml dispase (Sigma–Aldrich, St. Louis, MO, USA) for 45 min at room temperature (RT). Place the dispase digested lungs into 7 ml DMEM containing 25 mM Hepes and 100 U/ml DNaseI (Sigma–Aldrich) and cut lung tissues into small pieces and continue to digest for 10 min at RT. Mince the lungs and pass them through 100 and 40 μm cell strainer. Spin down the cells at 1600 rpm for 10 min and plate the cells into a 100 mm dish (previously coated with 42 μg CD45 antibody and 16 μg CD16/32 antibody) containing 10 ml DMEM with 10% fetal bovine serum (FBS) and 25 mM Hepes. Incubate the cells in a 37°C incubator for 45 min. The unattached cells were collected and cultured in DMEM supplemented with 10% FBS. Cells was identified by the pro‐SPC (marker of AECIIs) and FSP1 (marker of fibroblast) staining (Figure S1B).

Mouse AECIIs were transduced with lentivirus‐mediated PINK1 siRNA (5′‐gcGGTAATTGACTACAGCAAA‐3′) or negative control (5′‐TTCTCCGAACGTGTCACGT‐3′) lentivirus, and lentivirus‐mediated PINK1 overexpression (NM_026880), the lentiviruses were purchased from Genechem (Shanghai, China) at a multiplicity of infection of 50 according to the manufacturer's recommendations. For lentivirus‐mediated PINK1 siRNA the target sequence of the mouse PINK1 gene inserted is 5′‐gcGGTAATTGACTACAGCAAA‐3′. The lentivirus was produced with the vehicle components hU6‐MCS‐CMV‐Puromycin for siRNA and Ubi‐MCS‐SV40‐puromycin for overexpression. The detection of infection efficiency and the selection of transfected cells was determined using puromycin which was identified by qPCR (Figure S1C). The transduced AECIIs were treated with or without IL‐17A (14‐8171‐80, eBioscience, Waltham, MA, USA) (25 ng/ml) for 24 h followed by immunofluorescence co‐localization staining.

### 2.4 Mitochondrial membrane potential (MMP)

The changes in relative MMP were measured using the JC‐10 Mitochondrial Membrane Potential Assay Kit‐Microplate (ab112134, Abcam, Cambridge, UK) according to the manufacturer's instructions. Briefly, 5 × 10^4^ cells/well were cultured in 10% FBS‐DMEM in a 96‐well plate with a gradient concentration of IL‐17A (0, 10, 25, and 50 ng/ml) for 24 h. The cells were washed, the medium was changed to PBS, and JC‐10 dye was added. The cells were then incubated in a 37°C, 5% CO_2_ incubator for 30 min, protected from light. JC‐10‐labelled cells were monitored using a microplate reader for fluorescence intensities at excitation/emission = 520 nm/590 nm for ratio analysis.

### 2.5 Oxygen consumption rate (OCR)

OCR was measured using the Seahorse XF24 Extracellular Flux analyser (Seahorse Bioscience, Chicopee, MA, USA). Following treatment with IL‐17A (25 ng/ml) for 24 h, cells were rinsed with media, then washed and cultured in an XF assay medium supplemented with 1 mmol/L sodium pyruvate, 2.5 mmol/L glucose, and 4 mmol/L GlutaMax in a non‐CO_2_ incubator for 45 min. OCR was assessed through sequential injections of 1.5 μmol/L oligomycin, 2 μmol/L FCCP, and 0.5 μmol/L rotenone.

### 2.6 DNA extraction and ratio of mtDNA to genomic DNA (mtDNA/gDNA ratio)

Purification of DNA from lung tissues and primary mouse AECIIs was performed using All Prep DNA Kits (Qiagen, Dusseldorf, Germany) according to the manufacturer's recommendations. To quantify the mtDNA/gDNA ratio, qPCR was used to amplify one gene of the mouse mitochondrial genome (Nd2) and one gene of the mouse nuclear genome (GAPDH). Primer sequences were as follows (5′‐3′): GAPDH forward: CCTGCACCACCAACTGCTTAG; GAPDH reverse: GTGGATGCAGGGATGATGTTC; Nd2 forward: ATCCTCCTGGCCATCGTACT; Nd2 reverse: ATCAGAAGTGGAATGGGGCG.

### 2.7 Western blot analyses and immunofluorescence staining

Proteins from tissues or cultured cells were extracted, transferred onto nitrocellulose filter membranes (NC‐membranes), hybridized overnight with appropriate primary antibodies (DRP1‐ab184247, OPA1‐ab157457, MFN1‐ab221661, BAX‐ab32503 and 60267‐1‐lg, Bcl‐2‐ab182858 and 26593‐1‐AP, PINK1‐ab23707, Parkin‐66674‐1‐lg, and β‐actin‐66009‐1‐lg) obtained from Abcam (ab) or Proteintech (Chicago, IL, USA), and visualized using the Odyssey DLx Imaging System (Li‐Cor, Lincoln, NE, USA) or Gel Doc XR+ Imaging System (Bio‐rad, California, USA), according to the manufacturers protocol. Immunofluorescence analyses were performed using primary antibodies (pro‐SPC‐ab211326 and sc‐518029, IL‐17RA‐ab180904, ATP synthase C‐ab181243, BAX‐60267‐1‐lg, and PINK1‐sc‐517353) obtained from Abcam (ab), Proteintech, or Santa Cruz Biotechnology (sc, Dallas, Texas, USA). The secondary antibodies were IRDye 680RD goat anti‐mouse IgG (Li‐Cor, 926‐68070) and IRDye 680RD goat anti‐rabbit IgG (Li‐Cor, 926‐68071).

TUNEL assays were performed using the in situ Cell Death Detection Kit, Fluorescein (Roche, Basel, Switzerland, Catalogue No. 11684795910) according to the manufacturer's recommendations. DAPI staining was used to determine the number of nuclei and to assess gross cell morphology.

Immunofluorescence co‐localization staining was performed using Mito‐Tracker Red FM (M22425, Thermo Fisher Scientific, Waltham, MA, USA), a carbocyanine‐based dye that stains active mitochondria of AECIIs, according to the manufacturer's instructions. After fixation and blocking, the cells were incubated with a primary antibody against LC3 (Proteintech, 14600‐1‐AP), followed by an Alexa Fluor‐488 conjugated secondary antibody (abcam, ab150113). DAPI staining was used to determine the number of nuclei and to assess gross cell morphology.

### 2.8 Histopathology and transmission electron microscopy (TEM)

After the mice were sacrificed, the lungs were perfused with 4% paraformaldehyde (PFA), followed by paraffin embedding or saturation in 30% sucrose for 24 h for frozen sections. Paraffin sections were stained with haematoxylin and eosin or Masson trichrome staining to determine changes in histopathology and fibrosis, respectively. Immunohistochemistry studies used primary antibodies (fibronectin‐ab2413 and α‐SMA‐ab124964) obtained from Abcam. For TEM, mice were perfused with 4% PFA and lung samples were dissected out in 2% PFA, then fixed in 2.5% glutaraldehyde/2% PFA in 0.1 M sodium cacodylate buffer (pH 7.4). Samples were viewed using the Hitachi TEM system (Tokyo, Japan) at 80 kV. At least 10 cells from low‐ and high‐magnification images (6000× and 12,000×) were used to count the number of mitochondria per AECII (identified by the presence of lamellar bodies).

### 2.9 Statistical analysis

The number of samples in each experiment was at least 4 (*n* ≥ 4) and every experiment was repeated at least three times. All data are expressed as mean ± standard error of the mean (SEM). Data analyses were performed using a two‐tailed Student's *t*‐test or one‐way anova, followed by post‐hoc tests to determine pairs of groups, using GraphPad Prism 8 software (GraphPad, San Diego, CA, USA). A *p*‐value less than 0.05 was considered significant.

## 3 RESULTS

### 3.1 The expression of IL‐17RA and ATP synthase C is upregulated in AECIIs of IPF lungs

**FIGURE 1:**
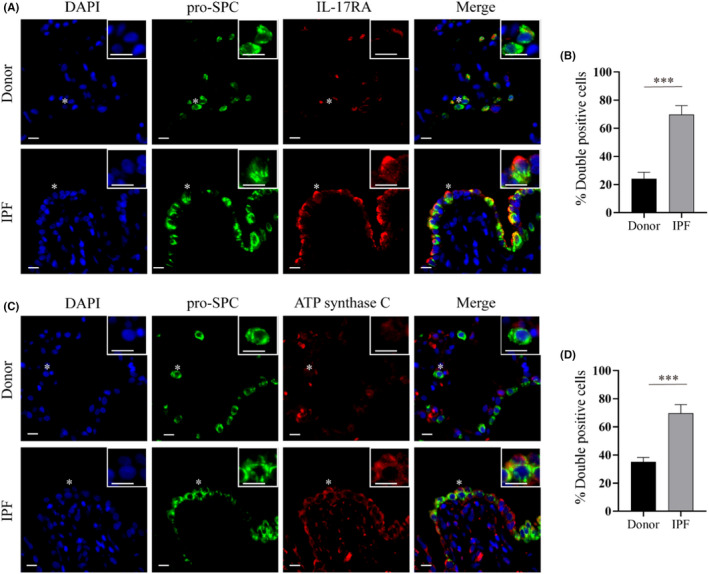
The expression of IL‐17RA and ATP synthase C is upregulated in AECIIs of IPF lungs. (A,C) Representative immunofluorescence images showing IL‐17RA (red) (A) and mitochondria (using ATP synthase C as a marker, red) (C) in AECIIs (using anti‐pro‐SPC as a marker, green) of donor control lungs (upper panels, *n* = 5 per group) and IPF lungs (lower panels, *n* = 8 per group). Representative cells (asterisks) are shown in detail in the insets. Scale bars: 10 μm. (B,D) Semiquantitative scoring of pro‐SPC/IL‐17RA double‐positive cells (B) and pro‐SPC/ATP synthase C double‐positive cells (D) as a percentage of total pro‐SPC‐stained cells. Data are presented as mean ± SEM. ***p < 0.001 versus donor

Because IL‐17A mainly acts by binding to IL‐17RA in vivo, the expression of IL‐17RA can represent the activity of IL‐17A signalling to a certain extent. We detected IL‐17RA expression and mitochondria levels in AECIIs by immunofluorescence colocalization analyses in consecutive lung sections of end‐stage IPF patients (*n* = 8, Table S1) and donor controls (*n* = 5) using prosurfactant protein C (pro‐SPC) and ATP synthase C as markers of AECIIs and mitochondria, respectively. The hyperplasic AECIIs from dense fibrotic areas of IPF lungs exhibited increased IL‐17RA expression levels compared with AECIIs from donor controls (Figure 1A,B). Noteworthily, at the same site of the IPF lungs, accumulated mitochondrial puncta were observed in these AECIIs that highly expressed IL‐17RA (Figure 1C,D). The abnormal accumulation of mitochondria in AECIIs of IPF lungs has been previously reported.^8^ These phenomena suggested a relationship between IL‐17A signals and mitochondrial accumulation of AECIIs in IPF lungs.

### 3.2 IL‐17A knockout alleviates BLM‐induced lung fibrosis, IL‐17RA upregulation, and mitochondrial abnormalities in AECIIs

**FIGURE 2:**
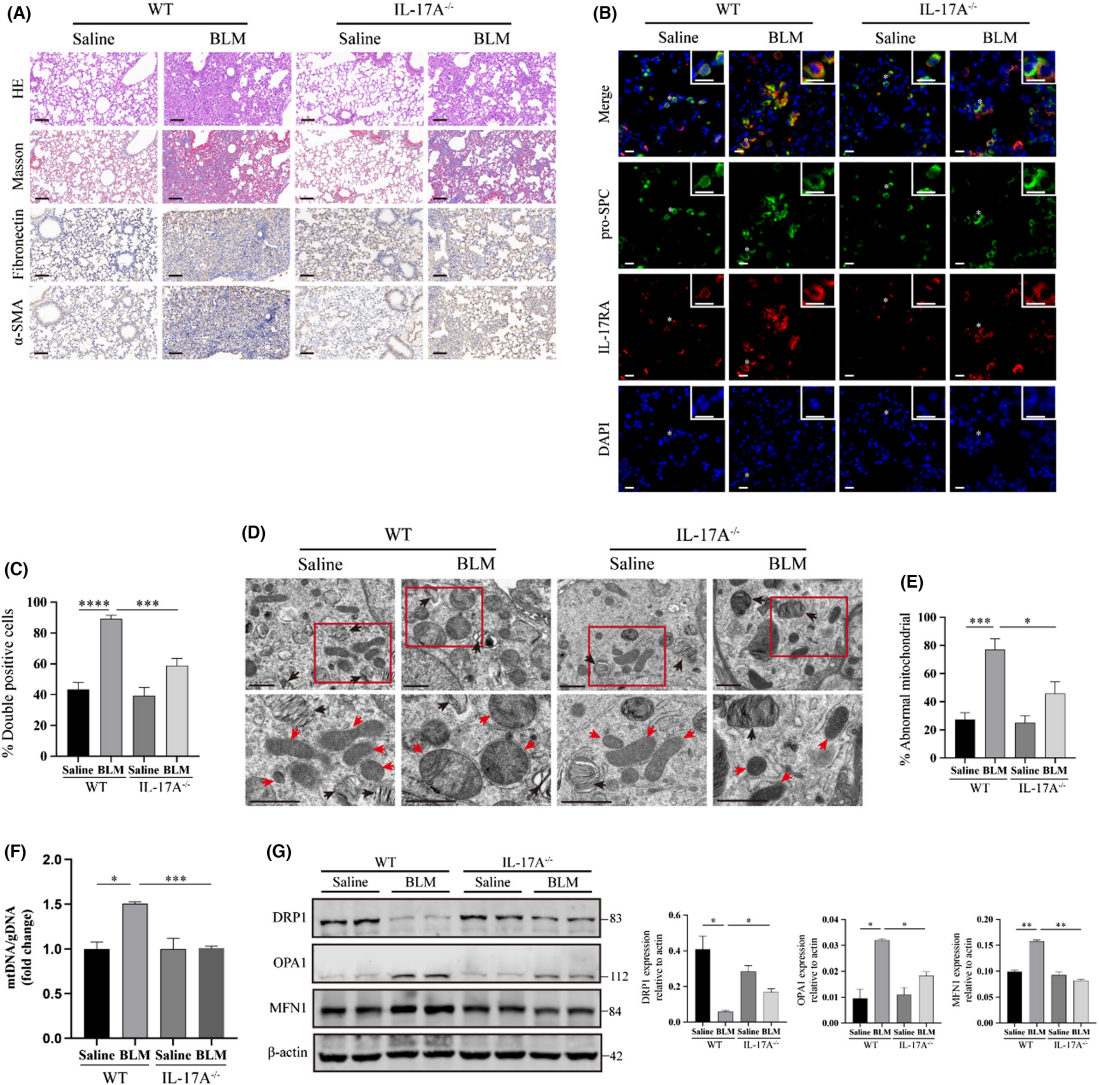
IL‐17A knockout alleviates BLM‐induced lung fibrosis, IL‐17RA upregulation, and mitochondrial abnormalities in AECIIs. (A) Haematoxylin and eosinstaining, Masson's Trichrome staining, and immunohistochemistry staining of fibronectin and α‐SMA of lung sections from WT and IL‐17A^−/−^ mice treated with saline or bleomycin (1.5 U/kg). Scale bars: 100 μm. (B) Representative immunofluorescence images showing IL‐17RA (red) in AECIIs (using anti‐pro‐SPC as a marker, green) of WT and IL‐17A^−/−^ mice treated with saline or bleomycin. Representative cells (asterisks) are shown in detail in the insets. Scale bars: 10 μm. (C) Semiquantitative scoring of pro‐SPC/IL‐17RA double‐positive cells as a percentage of total pro‐SPC‐stained cells. (D) Representative TEM images of AECIIs from wild‐type mice and IL‐17A^−/−^ mice challenged with BLM or saline. Boxed regions are shown enlarged in the lower panels. Black arrows indicate lamellar bodies, the characteristic organelles of AECIIs. Red arrows indicate mitochondria. Scale bars: 1 μm (upper panels), 500 nm (lower panels). (E) Quantitative analysis of the percentage of abnormal mitochondria, defined as damaged and swollen mitochondria with severely disrupted electron‐lucent cristae per cell/group. (F) mtDNA/gDNA in AECIIs isolated from lung samples showed mitochondrial mass after BLM or saline intratracheal injection in wild‐type mice and IL‐17A^−/−^ mice. (G) Western blot and quantitative analysis of markers of mitochondrial dynamics (DRP1, OPA1, and MFN1) in AECIIs isolated from lung samples derived from each group of mice indicated. Immunoblot gels were cropped and uncropped images of the immunoblot gels are in Figure S2A. Data are expressed as mean ± SEM. *p < 0.05, **p < 0.01, ***p < 0.001, ****p < 0.0001

To further determine the role of IL‐17A in PF and mitochondrial homeostasis in AECIIs, we administered BLM or saline intratracheal injection to IL‐17A knockout (IL‐17A^−/−^) mice and wild‐type (WT) mice. Lung histopathology collagen fibre staining of IL‐17A^−/−^ and WT mice was detected by haematoxylin and eosin staining and Masson trichrome staining at day 21 after injection. Lungs of IL‐17A^−/−^ mice showed decreased areas of interstitial fibrosis that correlated with lower expression of fibrotic extracellular matrix collagen, fibronectin, and α smooth muscle actin (α‐SMA) compared with WT mice (Figure 2A). To verify the changes of IL‐17RA expression in AECIIs from BLM‐induced PF mouse models, we also detected the IL‐17RA level of AECIIs by immunofluorescence colocalization analyses in lung sections of BLM or saline‐administered IL‐17A^−/−^ and WT mice using pro‐SPC as AECIIs marker. Similar to the IPF lungs, the IL‐17RA level of AECIIs from BLM‐induced PF lungs was significantly higher than that from saline controls, while AECIIs from IL‐17A^−/−^ lungs showed decreased IL‐17RA expression compared with that from WT lungs (Figure 2B,C).

Ultrastructural pictures photographed by TEM demonstrated that AECIIs (marked by the lamellar body, the characteristic organelle of AECIIs) from IL‐17A^−/−^ mice after BLM damage showed a decreased frequency of enlarged and dysmorphic mitochondria, defined as swollen mitochondria with severely disrupted electron‐lucent cristae, compared with AECIIs from BLM‐injured WT mice (Figure 2D,E). Moreover, after BLM treatment, primary AECIIs isolated from the lungs of IL‐17A^−/−^ mice showed lower mtDNA levels compared with those of WT mice (Figure 2F). The mtDNA/gDNA ratio represents the content of mitochondria on one hand, and is associated with mitochondrial homeostasis on the other hand because mtDNA has higher DNA mutation rates than that of nuclear DNA.^20^ Mitochondrial enlargement and dysmorphia could result from the dysregulation of mitochondrial dynamics including assembly (fusion) and disassembly (fission), which also influence a variety of cellular biological processes such as apoptosis.^21^ Western blot analyses showed disrupted mitochondrial dynamics in primary AECIIs isolated from BLM‐induced fibrotic lungs with lower expression of dynamin‐related protein 1 (DRP1), which is a central regulator of the canonical mitochondrial fission machinery.^22^ There were increased expression levels of optic atrophy 1 (OPA1) and mitofusin 1 (MFN1), which are key dynamin‐related proteins controlling mitochondrial fusion,^22^ while IL‐17A knockout improved this dysregulation of mitochondrial dynamics injured by BLM (Figure 2G).

### 3.3 IL‐17A impairs mitochondrial function and fusion/fission dynamics in AECIIs

**FIGURE 3:**
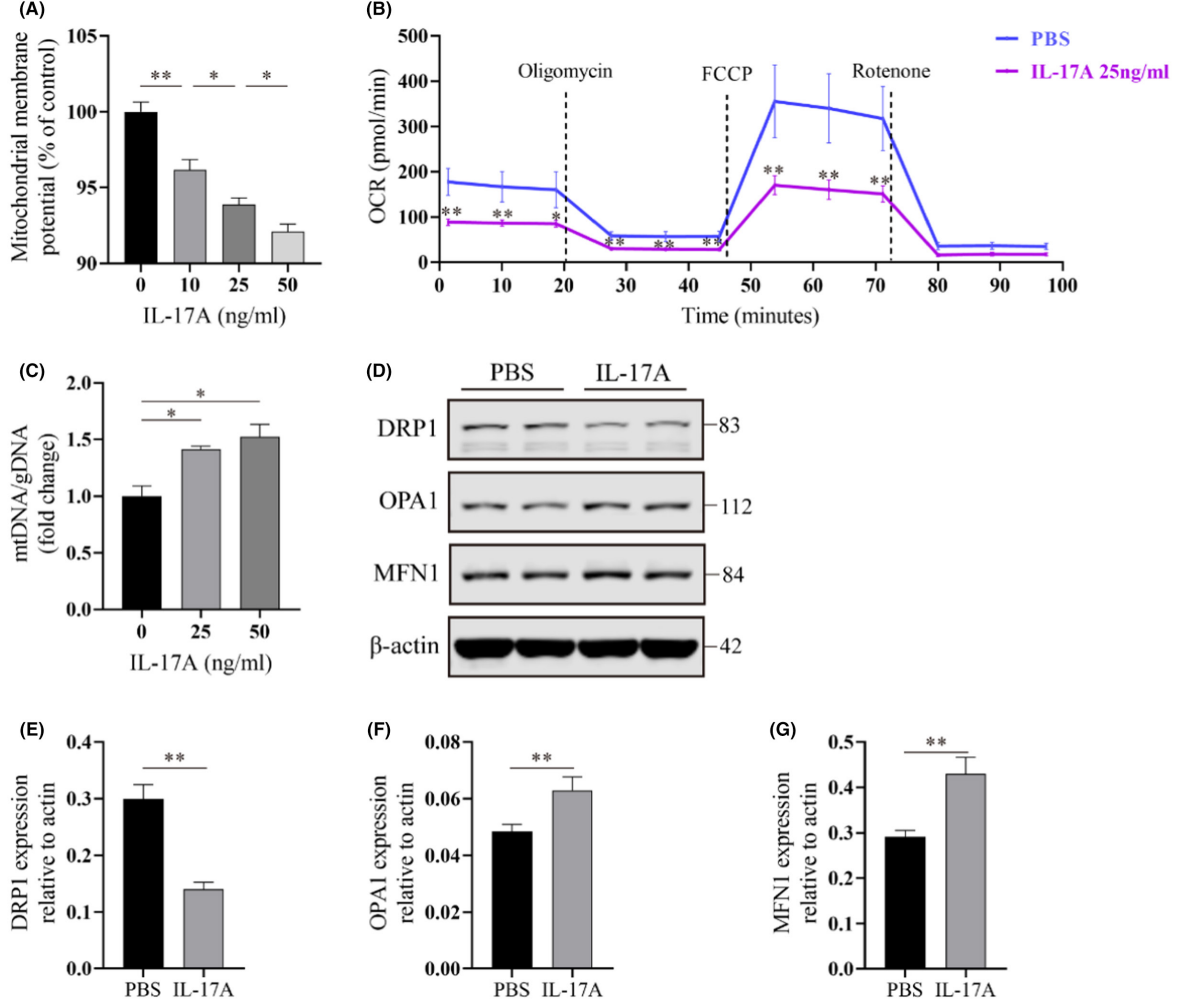
IL‐17A impairs mitochondrial function and fusion/fission dynamics in AECIIs. (A) Fluorescence intensities at excitation/emission = 520 nm/590 nm ratio as a readout of mitochondrial membrane potential in primary mice AECIIs with a gradient concentration of IL‐17A (0, 10, 25, and 50 ng/ml) stimulation for 24 h. (B) OCR (pmol/min) of AECIIs with or without IL‐17A treatment (25 ng/ml) for 24 h, as measured under basal conditions, followed by the addition of oligomycin (1.5 μmol/L), FCCP (2 μmol/L), as well as rotenone and antimycin (0.5 μmol/L), as indicated. (C) Mitochondrial mass, assessed by mtDNA/gDNA ratio, in AECIIs with IL‐17A treatment (0, 25, and 50 ng/ml) for 24 h. (D–G) Western blot (D) and quantitative analysis (E–G) of markers of mitochondrial dynamics (DRP1, OPA1, and MFN1) in AECIIs with or without IL‐17A treatment (25 ng/ml) for 24 h. Immunoblot gels were cropped and uncropped images of the immunoblot gels are in Figure S2B. Data are expressed as mean ± SEM. *p < 0.05, **p < 0.01

After showing that IL‐17A knockout blunted mitochondrial morphology abnormalities in primary AECIIs in vivo, we further assessed whether IL‐17A could impair the mitochondrial function of lung epithelial cells. We treated mouse primary AECIIs with a gradient concentration of IL‐17A and then measured MMP using the potentiometer dye JC‐10. We found that IL‐17A treatment diminished MMP in a concentration‐dependent manner (Figure 3A). To evaluate the effect of IL‐17A on mitochondrial respiration of AECIIs, we detected the OCR and observed a decreased basal, minimal (after treatment with the ATP synthase inhibitor oligomycin), and maximal (after treatment with the mitochondrial oxidative phosphorylation uncoupling agent FCCP) OCR when stimulated with IL‐17A (25 ng/ml) (Figure 3B). The reduction between baseline and minimum and the increase between baseline and maximum respectively represent oxygen consumption coupled to ATP production and oxygen consumption uncoupled mitochondrial respiration. The latter is the so‐called reserve capacity of mitochondrial respiratory function. We then detected the mtDNA/gDNA ratio, and IL‐17A significantly increased the mtDNA/gDNA ratio in AECIIs (Figure 3C). Western blot analyses of mitochondrial dynamic modulators in AECIIs showed that IL‐17A treatment decreased the protein expression of mitochondrial fission regulator DRP1 and increased the expression of mitochondrial fusion regulators OPA1 and MFN1 (Figure 3D–G). These findings suggest that IL‐17A can impair mitochondrial function and change mitochondrial dynamics to a fusion trend.

### 3.4 IL‐17A induces apoptosis of AECIIs both in vivo and in vitro

**FIGURE 4:**
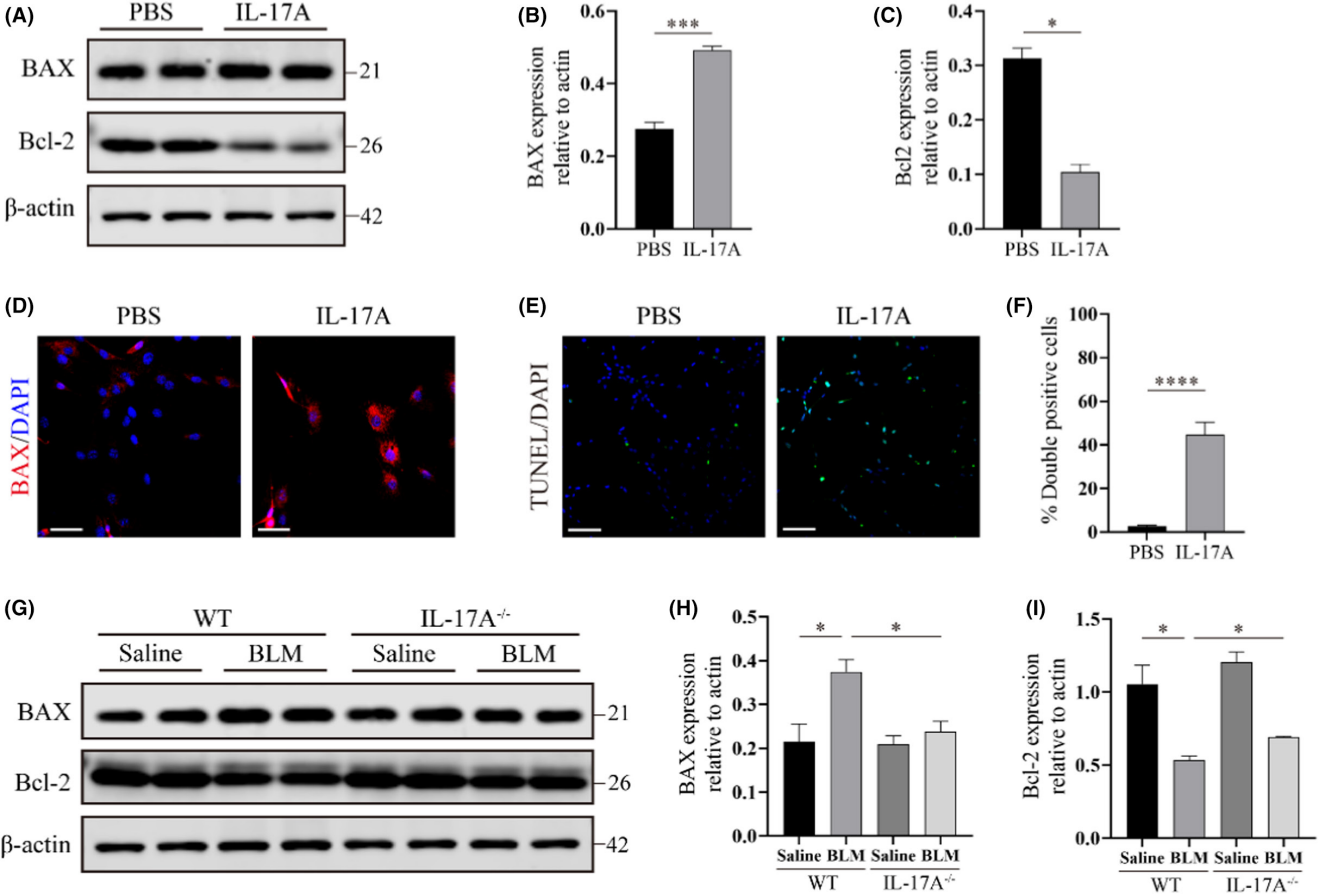
IL‐17A induces apoptosis of AECIIs both in vivo and in vitro. (A–C) Western blot analyses (A) and quantitative analysis (B,C) of markers of mitochondria cell apoptosis (BAX, Bcl‐2) in AECIIs with or without IL‐17A treatment (25 ng/ml) for 24 h. Immunoblot gels were cropped and uncropped images of the immunoblot gels are in Figure S2C. (D) Immunofluorescence analysis for BAX (red) in AECIIs with or without IL‐17A treatment (25 ng/ml) for 24 h. Scale bars: 50 μm. (E,F) Immunofluorescence (E) and quantitative analysis (F) of double‐positive AECIIs (TUNEL/DAPI) with or without IL‐17A treatment (25 ng/ml) for 24 h. Scale bars: 100 μm. (G–I) Western blot analyses (G) and quantitative analysis (H,I) of markers of mitochondria cell apoptosis (BAX, Bcl‐2) in AECIIs isolated from lung samples derived from each group of mice indicated. Immunoblot gels were cropped and uncropped images of the immunoblot gels are in Figure S2D. The data are expressed as mean ± SEM (mean percentage of double positive cells + SEM). *p < 0.05, ***p < 0.001, ****p < 0.0001

We next examined the effect of IL‐17A on apoptosis of AECIIs, as mitochondrial dysfunction can induce cell apoptosis.^23^ Western blot data revealed that IL‐17A treatment led to higher protein expression of the pro‐apoptotic mediator Bcl‐2 associated X protein (BAX) and lower expression of the anti‐apoptotic mediator Bcl‐2 in AECIIs (Figure 4A–C). Immunofluorescence assay results also showed that the fluorescence intensity of BAX protein staining in AECIIs increased after IL‐17A stimulation (Figure 4D). BAX upregulation and Bcl‐2 downregulation were related to enhanced apoptotic activity, as evidenced by a larger number of TUNEL‐positive cells (Figure 4E,F). This observation was verified in vivo by western blot analysis, which showed that AECIIs isolated from the bleomycin‐injured lungs of IL‐17A^−/−^ mice had significantly decreased BAX expression levels and increased Bcl‐2 expression levels compared with AECIIs isolated from the lungs of BLM‐treated WT mice (Figure 4G–I). These findings indicate that IL‐17A knockout can alleviate BLM‐induced apoptosis of AECIIs, consistent with the in vitro results.

### 3.5 IL‐17A inhibits mitophagy and promotes apoptosis of AECIIs by decreasing PINK1 signal expression

**FIGURE 5:**
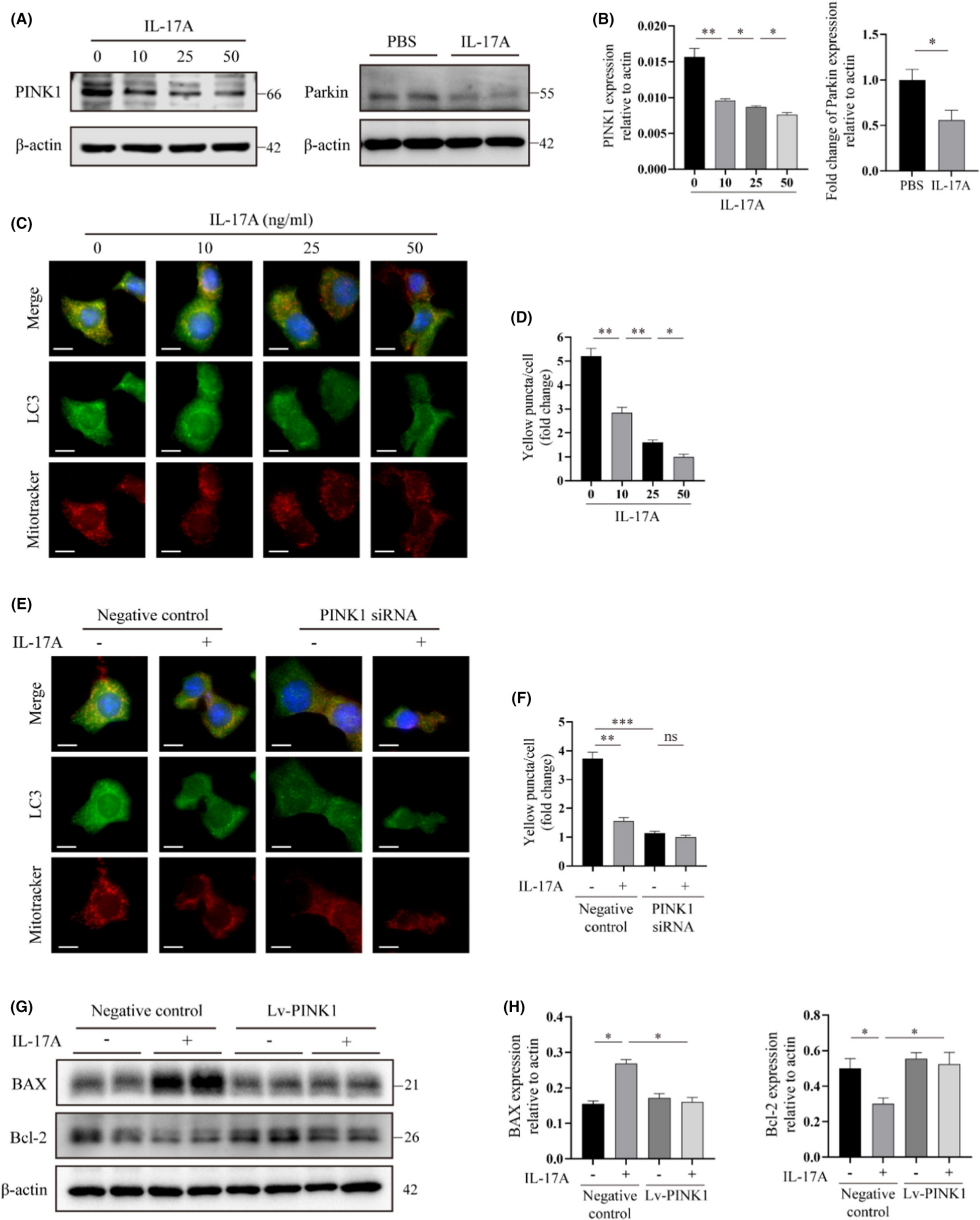
Il‐17A inhibits mitophagy and promotes apoptosis of AECIIs by decreasing PINK1 signal expression. (A, B) Western blot (A) and quantitative analysis (B) of mitophagy mediators PINK1 and Parkin in AECIIs with different concentrations of IL‐17A (0, 10, 25, and 50 ng/ml for PINK1 and 25 ng/ml for Parkin) stimulation for 24 h. Immunoblot gels were cropped and uncropped images of the immunoblot gels are in Figures S2E, S3A. (C, D) (C) Immunofluorescence co‐localization staining of Mito‐Tracker (a red cationic dye that stains mitochondria) and LC3 (green) in AECIIs after a gradient concentration of IL‐17A (0, 10, 25, and 50 ng/ml) stimulation for 24 h. The co‐localization of LC3 with Mito‐Tracker is indicated in yellow. Scale bars: 10 μm. (D) Semiquantitative scoring of yellow puncta per cell. (E, F) (E) Immunofluorescence co‐localization staining of Mito‐Tracker (red) and LC3 (green) in AECIIs transduced with lentivirus‐mediated PINK1 siRNA or the negative control lentivirus. The transduced cells were treated with or without IL‐17A (25 ng/ml) for 24 h. Localization of LC3 with Mito‐Tracker is indicated in yellow. Scale bars: 10 μm. (F) Semiquantitative scoring of yellow puncta per cell. (G,H) Western blot (G) and quantitative analysis (H) of markers of mitochondria cell apoptosis (BAX, Bcl‐2) in AECIIs transduced with lentivirus‐mediated PINK1 overexpression or the negative control lentivirus. The transduced cells were treated with or without IL‐17A (25 ng/ml) for 24 h. Immunoblot gels were cropped and uncropped images of the immunoblot gels are in Figure S3B. Data are presented as mean ± SEM. *p < 0.05, **p < 0.01, ***p < 0.001

PINK1 is a key mediator of mitochondrial quality control, and its deficiency diminishes the ability of mice to degrade dysfunctional mitochondria by autophagy (mitophagy), thus increasing the susceptibility to PF.^8^ Parkin is a maintain downstream regulatory protein of PINK1, which ubiquitinates mitochondrial proteins to form a ubiquitin amplification loop that eventually initiates mitophagy.^24^ We detected PINK1 and Parkin protein levels in AECIIs after IL‐17A stimulation by western blot analysis. The results showed that IL‐17A‐treated AECIIs had lower levels of PINK1 and Parkin expression than that PBS‐treated (Figure 5A,B). To further determine the levels of intracellular mitophagy, we next performed co‐localization staining by labeling mitochondria and light chain 3 (LC3) proteins in AECIIs with a mitochondrial red fluorescent probe (Mitotracker) and green fluorescent protein, respectively. We observed that IL‐17A treatment also inhibited the mitophagy levels in AECIIs in a concentration‐dependent manner, as manifested by a decreased yellow fluorescence intensity after merging LC3 with mitochondria (Figure 5C,D).

We then used small interfering RNAs (siRNAs) mediated by lentivirus to silence the expression of PINK1 to further explore the relationship between IL‐17A‐associated effects on mitophagy in AECIIs and PINK1. Immunofluorescence co‐localization staining of mitochondria and LC3 proteins showed that siRNA‐mediated knockdown of PINK1 could decrease the mitophagy levels in AECIIs compared with the negative control. Additionally, after being transfected with PINK1 siRNA, mitophagy in AECIIs could not be further reduced by IL‐17A treatment (Figure 5E,F). To determine the relationship between the effect of IL‐17A on promoting apoptosis and PINK1, the method of lentivirus transfection was used to overexpress PINK1 in AECIIs. The results indicated that the pro‐apoptotic effect of IL‐17A was significantly attenuated after PINK1 overexpression, manifested as the decrease of BAX protein and the increase of Bcl‐2 protein (Figure 5G,H).

**FIGURE 6:**
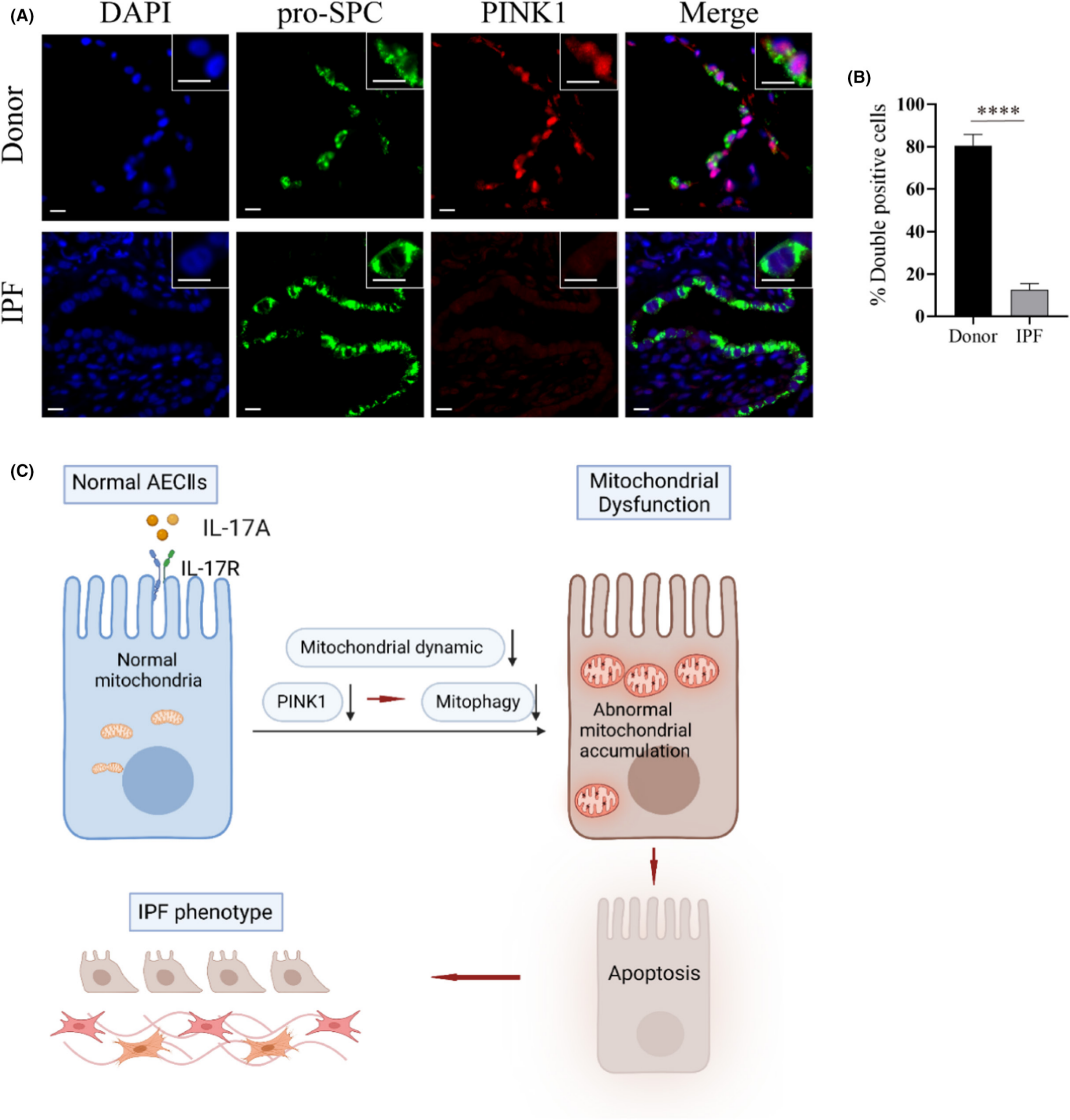
Decreased PINK1 expression in AECIIs of IPF lungs, and a schematic presentation of the mechanism of this study. (A) Representative immunofluorescence images showing PINK1 (red) in AECIIs (using anti‐pro‐SPC as a marker, green) of donor control lungs (upper panels, *n* = 5 per group) and IPF lungs (lower panels, *n* = 8 per group). Representative cells (asterisks) are shown in detail in the insets. Scale bars: 10 μm. (B) Semiquantitative scoring of pro‐SPC/PINK1 double‐positive cells as a percentage of total pro‐SPC‐stained cells. Data are presented as mean ± SEM. ****p < 0.0001. (C) A schematic presentation of the mechanism by which IL‐17A causes mitochondrial accumulation and mitochondrial dysfunction of AECIIs possibly through disturbing mitochondrial dynamics, decreasing the expression of PINK1, and inhibiting mitophagy. This induces apoptosis of AECIIs and ultimately promotes pulmonary fibrosis

We then detected PINK1 protein expression in AECIIs from donor controls and IPF lungs by immunofluorescence assays. PINK1 staining was lower in AECIIs from fibrotic areas in IPF lungs compared with those of donor controls (Figure 6A,B), suggesting that PINK1 expression is dysregulated in IPF.

## 4 DISCUSSION

Transcriptomic analyses of IPF epithelial cells identified the changes in metabolic pathways of AECIIs, which include alterations in mitochondrial biomass or function.^25^, ^26^ Additionally, the effects of AECIIs in recruiting effector immune cells such as macrophages and monocytes are considered to be a crucial part of lung fibrogenesis.^27^, ^28^ Recently, a strong pro‐inflammatory cytokine IL‐17A has been proven to promote lung fibrosis, while the mechanism is unclear.^29^, ^30^, ^31^ Our study showed that IL‐17A could induce disordered mitochondrial dynamics and impaired mitophagy, thereby leading to mitochondria dysfunction in AECIIs and further promoting apoptosis of these cells, which elucidated a novel mechanism of IL‐17A in the pathogenesis of IPF.

IL‐17R is widely distributed on the surface of various types of cells, including epithelial cells and fibroblasts in the lung, allowing IL‐17A to act as an important mediator in the pathophysiology of chronic respiratory diseases.^32^ Polarized airway epithelial cells respond to IL‐17A via the basolateral expression of IL‐17RA^33^ and IL‐17RC.^34^ Using a lung epithelial specific conditional IL‐17RA KO mice, Kong et al.^35^ proved that in the lung epithelium IL‐17A played a critical role in bronchiolar mucosal immunity against pulmonary bacterial pathogens through IL‐17R signalling. However, during PF, Su et al. reported that IL‐17A promoted the mesenchymal transition of alveolar epithelial cells in a TGF‐β1‐dependent manner and attenuated the autophagy as well as autophagy‐mediated cell death in the alveolar epithelial cells.^36^ Moreover, another study indicated that inhibiting IL‐17A could alleviate LPS‐induced lung injury via decreasing NF‐κB infiltration of dendritic cells and endoplasmic reticulum (ER) stress in bronchial epithelial cells.^37^ They also found that the expression of both IL‐17A protein and IL‐17A mRNA increased after LPS stimulated in bronchial epithelial cells, which suggested that airway epithelial cells could also be a source of IL‐17A in addition to its target.

It has been reported that the expression of IL‐17RA is upregulated in RA‐ILD and IPF lungs compared with the healthy control.^38^ What is more, incubation of human lung fibroblasts with IL‐17A also induces IL‐17RA upregulation over time.^38^ In our study, to explore the mechanism of IL‐17A involvement in IPF, we focused on the alveolar epithelial cells, which were identified as initiating cells of IPF. We found that IL‐17RA expression levels in AECIIs were significantly increased in IPF lung tissues as well as in BLM‐induced PF mice, suggesting an enhanced IL‐17A immune response in the process of PF. In addition to that, ATP synthase C, a marker of mitochondria, was also increased in AECIIs at the same sites of IPF lung samples. These observations indicate that the accumulation of mitochondria in AECIIs of IPF is possibly associated with the effects of IL‐17A. As previously mentioned, IL‐17A could increase the level of ER stress in airway epithelial cells.^37^ Persistent ER stress impairs mitochondria bioenergetics and leads to mitochondrial swelling,^39^ which induces mitochondrial dysfunction of AECIIs in PF.^8^ Thus, we established a PF murine model and observed that, in addition to alleviating PF as previously reported,^14^ IL‐17A knockout also attenuated BLM‐induced mitochondrial morphological abnormalities in AECIIs. We then stimulated the mouse primary AECIIs with exogenous IL‐17A and observed significant disorders of mitochondrial quality control, including mitochondrial membrane depolarization, diminished energy production, and an increased mtDNA/gDNA ratio. Owing to the greater replication rates inducing inherent errors as well as the proximity of mtDNA to electron transport‐generated oxyradicals, mtDNA has higher DNA mutation rates than that of nuclear DNA.^20^ The higher ratio of mtDNA/gDNA has been recognized as damage‐associated molecular patterns because it is related to almost all aspects of mitochondrial quality control, including the mitochondrial unfolded protein response (UPRmt), inflammation, apoptosis and mitophagy.^20^ Moreover, circulating mtDNA has recently been reported as a promising biomarker in IPF.^40^

Mitochondria are highly dynamic organelles that require a balance between fission and fusion for proper functioning and adaptation to cell growth, division, and injury response.^41^, ^42^ In AECIIs, consequences of mitochondrial dynamics destruction include energy production impairment, oxidative stress damage, and mtDNA release.^43^, ^44^ Here, we found decreased protein levels of mitochondrial fission regulator DRP1 and increased levels of mitochondrial fusion mediators OPA1 and MFN1 in mouse AECIIs after IL‐17A stimulation in vitro, which was consistent with the findings in vivo. This suggests that IL‐17A impairs mitochondrial dynamics in AECIIs, specifically by inhibiting fission, promoting fusion, and making mitochondria unable to self‐renew and gradually accumulate. Apoptosis of epithelial cells is increasingly recognized as a hallmark of PF.^45^ Mitochondrial‐induced apoptosis is one of the mechanisms of innate immunity that is initiated by BAX and other pro‐apoptotic mediators penetrating the mitochondrial outer membrane.^20^ In our study, we demonstrated that after being exposed to IL‐17A, AECIIs exhibited an increased level of apoptosis, while knockout of IL‐17A could attenuate BLM‐induced AECII apoptosis.

AECIIs selectively remove individual subcellular components, such as invading pathogens, lipids, and dysfunctional cellular organelles, through autophagy. Inhibition of autophagy promotes epithelial‐fibrosis crosstalk, which contributes to fibrosis.^46^ The degradation of dysfunctional mitochondria is termed mitophagy, which is part of a large cellular system that maintains the homeostasis of mitochondria.^47^ Impairments in mitophagy have been associated with multiple diseases, including age‐related PF.^48^, ^49^ One of the main steps of mitophagy is the stabilization of PINK1 in response to the lowered transmembrane potential of damaged mitochondria.^20^ Decreased expression of PINK1 and perturbations in mitophagy are observed in AECIIs of IPF.^50^ Chemically induced AECII ER stress produces a decrease in PINK1 levels, thus linking a wider dysfunction in AECII quality control of malformed proteins with impaired mitochondrial homeostasis in IPF.^51^ Here, we observed that IL‐17A could induce both the decrease of PINK1/Parkin signal expression and mitophagy level in AECIIs. Silencing PINK1 mRNA expression in AECIIs could prevent the mitophagy‐inhibiting effect of IL‐17A. Furthermore, overexpression of PINK1 reversed IL‐17A‐induced apoptosis of AECIIs. Therefore, our data suggest that IL‐17A impairs mitophagy by reducing PINK1 levels which, together with the disturbance of mitochondrial dynamics, results in abnormal accumulation of mitochondria in AECIIs and eventually promotes cell death.

The mechanisms regarding IL‐17A in PF, inflammation, or lung cancer include a variety of signalling pathways, such as TGF‐β, PD‐1/STAT3, JAK2, and NF‐κB signalling. These have been shown to influence the pathophysiological processes of epithelial‐mesenchymal crosstalk, apoptosis, and autophagy of epithelial cells.^14^, ^36^, ^38^, ^52^, ^53^ Our work elucidated a new mechanism of IL‐17A in PF from an energy metabolism disorder perspective, which enriched and complemented the current research. In the clinic, exploring novel treatment methods containing combination therapies is a priority strategy to halt or reverse IPF. Our study provides a theoretical basis for the combined therapeutic strategy of antagonizing IL‐17A and targeting mitochondrial quality control of AECIIs to be applied in IPF. Admittedly, in this study, the clinical lung tissue samples we used had age bias because IPF patients who received lung transplantations were generally elderly people and the donors were generally younger people who died unexpectedly. We also did not explore the role of IL‐17A on mitochondrial biogenesis which played a role in the mitochondrial quality control machinery. Nevertheless, Rakhee et al. reported that IL‐17A stimulation markedly decreased the PGC1α expression in bronchial fibroblasts isolated from severe asthma patients, which indicated that IL‐17A impaired the mitochondrial biogenesis in lung fibroblasts.^17^

In summary, this study suggests that IL‐17A can impair the mitochondrial dysfunction of AECIIs by possibly disrupting mitochondrial dynamics, decreasing PINK1 expression, and inhibiting mitophagy. This promotes AECII apoptosis and strengthens the susceptibility to PF. Our work provides support for the development of potential therapies targeting IL‐17A and mitochondrial quality control to blunt PF, especially IPF.

## AUTHOR CONTRIBUTIONS

**Huijuan Xiao:** Conceptualization (equal); data curation (lead); formal analysis (equal); investigation (equal); methodology (equal); project administration (equal); resources (equal); software (lead); validation (equal); visualization (lead); writing – original draft (lead); writing – review and editing (equal). **Liang Peng:** Conceptualization (supporting); data curation (equal); formal analysis (equal); visualization (equal); writing – review and editing (equal). **Dingyuan Jiang:** Conceptualization (lead); data curation (equal); methodology (equal); writing – review and editing (equal). **Yuan Liu:** Resources (equal); software (equal). **Lili Zhu:** Resources (supporting). **Zhen Li:** Methodology (supporting); resources (supporting); software (supporting). **Jing Geng:** Resources (supporting). **Bingbing Xie:** Resources (supporting). **Xiaoxi Huang:** Conceptualization (supporting); resources (equal); writing – review and editing (supporting). **Jing Wang:** Conceptualization (supporting); data curation (supporting); writing – review and editing (supporting). **Huaping Dai:** Conceptualization (lead); data curation (equal); funding acquisition (lead); supervision (lead); writing – review and editing (lead). **Chen Wang:** Conceptualization (supporting); data curation (supporting); funding acquisition (supporting); supervision (lead); writing – review and editing (equal).

## CONFLICT OF INTEREST

The authors declare that they have no conflict of interest.

## Supporting information


Appendix S1

## Data Availability

The data that support the findings of this study are available from the corresponding author upon reasonable request.
