# Supplementary material for: IL‐17A promotes lung fibrosis through impairing mitochondrial homeostasis in type II alveolar epithelial cells
Source: J Cell Mol Med. 2022 Oct 29;26(22):5728–41. doi: 10.1111/jcmm.17600 (PMC9667517; doi:10.1111/jcmm.17600)
Supplement: Supplementary file 1 — Appendix S1 [file JCMM-26-5728-s001.docx]

Supplemental Materials

IL-17A promotes lung fibrosis through impairing mitochondrial homeostasis in type II alveolar epithelial cells

Huijuan Xiao^1,2^, Liang Peng^3^, Dingyuan Jiang^2^, Yuan Liu^2,4^, Lili Zhu^2^, Zhen Li^2^, Jing Geng^2^, Bingbing Xie^2^, Xiaoxi Huang^5^, Jing Wang^6^, Huaping Dai^2*^ and Chen Wang^1,2*^

^1^Department of Pulmonary and Critical Care Medicine, Center of Respiratory Medicine, China-Japan Friendship Hospital, School of Clinical Medicine, Peking University, Beijing, China;

^2^Department of Pulmonary and Critical Care Medicine, Center of Respiratory Medicine, China-Japan Friendship Hospital; National Center for Respiratory Medicine; National Clinical Research Center for Respiratory Diseases; Institute of Respiratory Medicine, Chinese Academy of Medical Sciences, Peking Union Medical College; Beijing, China;

^3^Beijing Key Laboratory for Immune-Mediated Inflammatory Diseases, Institute of Medical Science, China-Japan Friendship Hospital, Beijing, China;

^4^Department of Respiratory and Critical Care Medicine, Zhongnan Hospital of Wuhan University, Wuhan, China;

^5^Medical Research Center, Beijing Chaoyang Hospital Affiliated to Capital Medical University, Beijing, China;

^6^State Key Laboratory of Medical Molecular Biology, Department of Physiology, Institute of Basic Medical Sciences Chinese Academy of Medical Sciences, School of Basic Medicine Peking Union Medical College, Beijing, China.

*Correspondence:

Department of Pulmonary and Critical Care Medicine, China-Japan Friendship Hospital, No. 2 Yinghua East Road, Chaoyang District, Beijing 100029, China

Email: [daihuaping@ccmu.edu.cn](mailto:daihuaping@ccmu.edu.cn) (H D), cyh-birm@263.net (C W)

**Supplementary table. Demographics characteristics of patient cohort.**

|  | **IPF** | **Donor-Control** |
| --- | --- | --- |
| **Subjects** | 8 | 5 |
| **Age (yr) (Mean + SD)** | 63.50+ 3.665 | 30.20+ 10.06 |
| **Gender** |  |  |
| Males | 7 (87.5%) | 3 (60%) |
| Females | 1 (12.5%) | 2 (40%) |


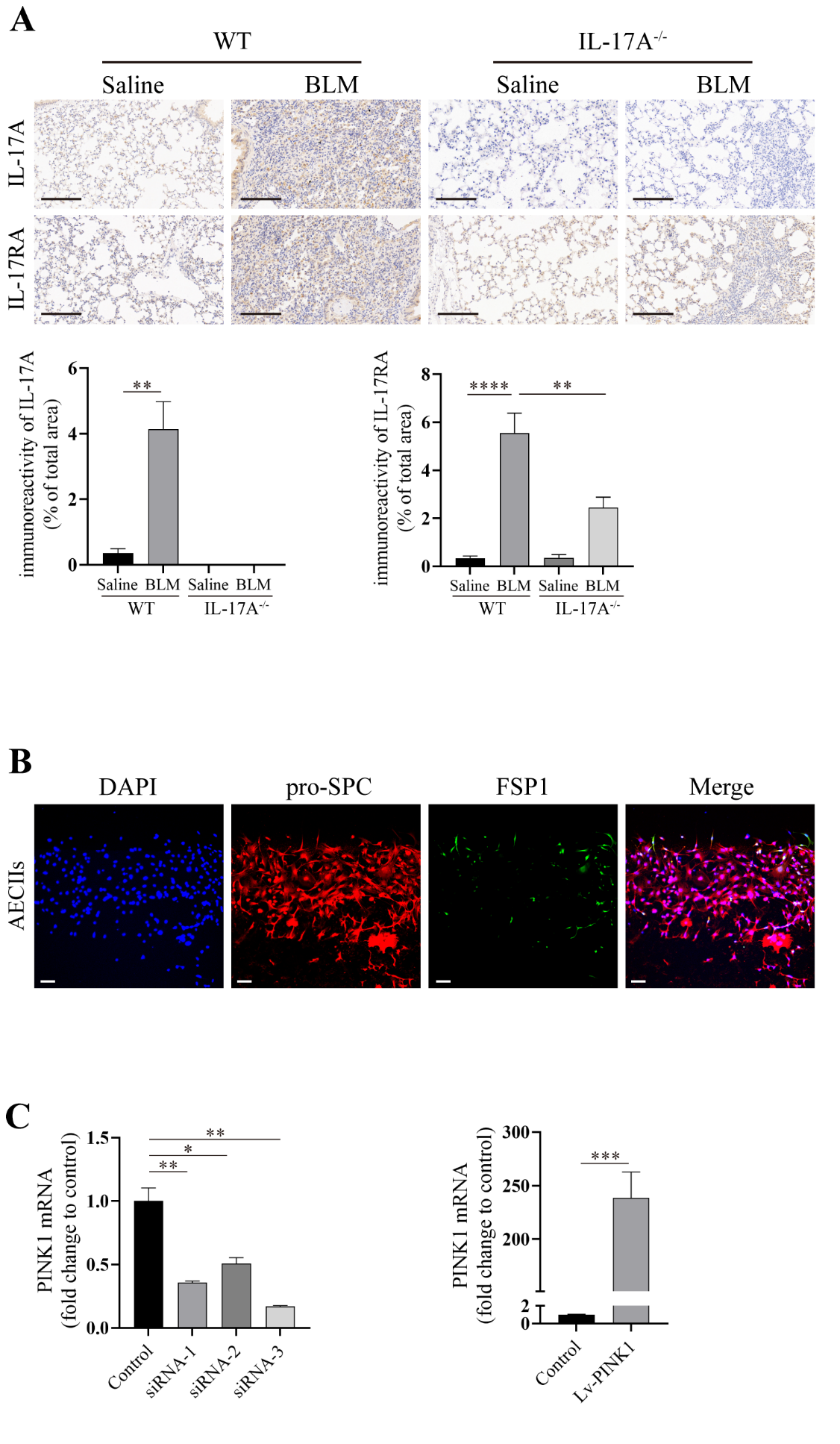


**Supplementary figure 1. (A)** Immunohistochemistry staining and quantitative analysis of IL-17A and IL-17RA of lung sections from WT and IL-17A-/- mice treated with saline or bleomycin (1.5 U/kg). Scale bars: 100μm. **(B)** Representative immunofluorescence images showing pro-SPC (red, marker of AECIIs) and FSP1 (green, marker of fibroblast) of mice primary AECIIs isolated. Scale bars: 100μm. **(C)** The expression of PINK1 mRNA in AECIIs transduced with lentivirus-mediated PINK1 siRNA or the negative control lentivirus (left panel, we chose virus strain 2 for the final experiment) and lentivirus-mediated PINK1overexpression or the negative control lentivirus (right panel). Data are presented as mean ± SEM. **P* < 0.05, ***P* < 0.01, ****P* < 0.001, *****P* < 0.0001.


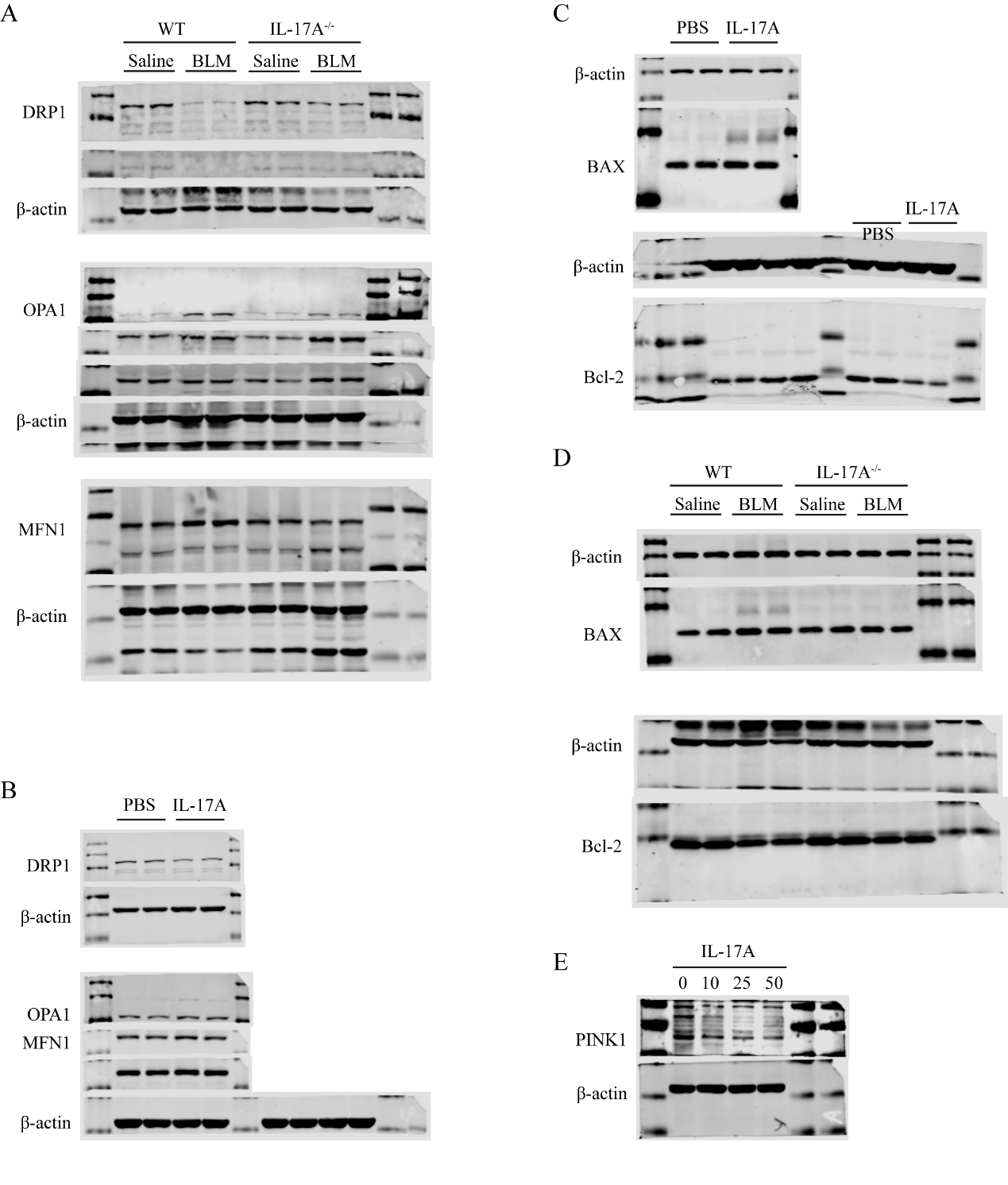


**Supplementary figure 2. Uncropped images of the immunoblot gels.**

(**A**) Uncropped images for figure 2. (**B**) Uncropped images for figure 3. (**C**) Uncropped images for figure 4 (AECIIs in vitro). (**D**) Uncropped images for figure 4 (AECIIs in vivo). (**E**) Uncropped images for figure 5.


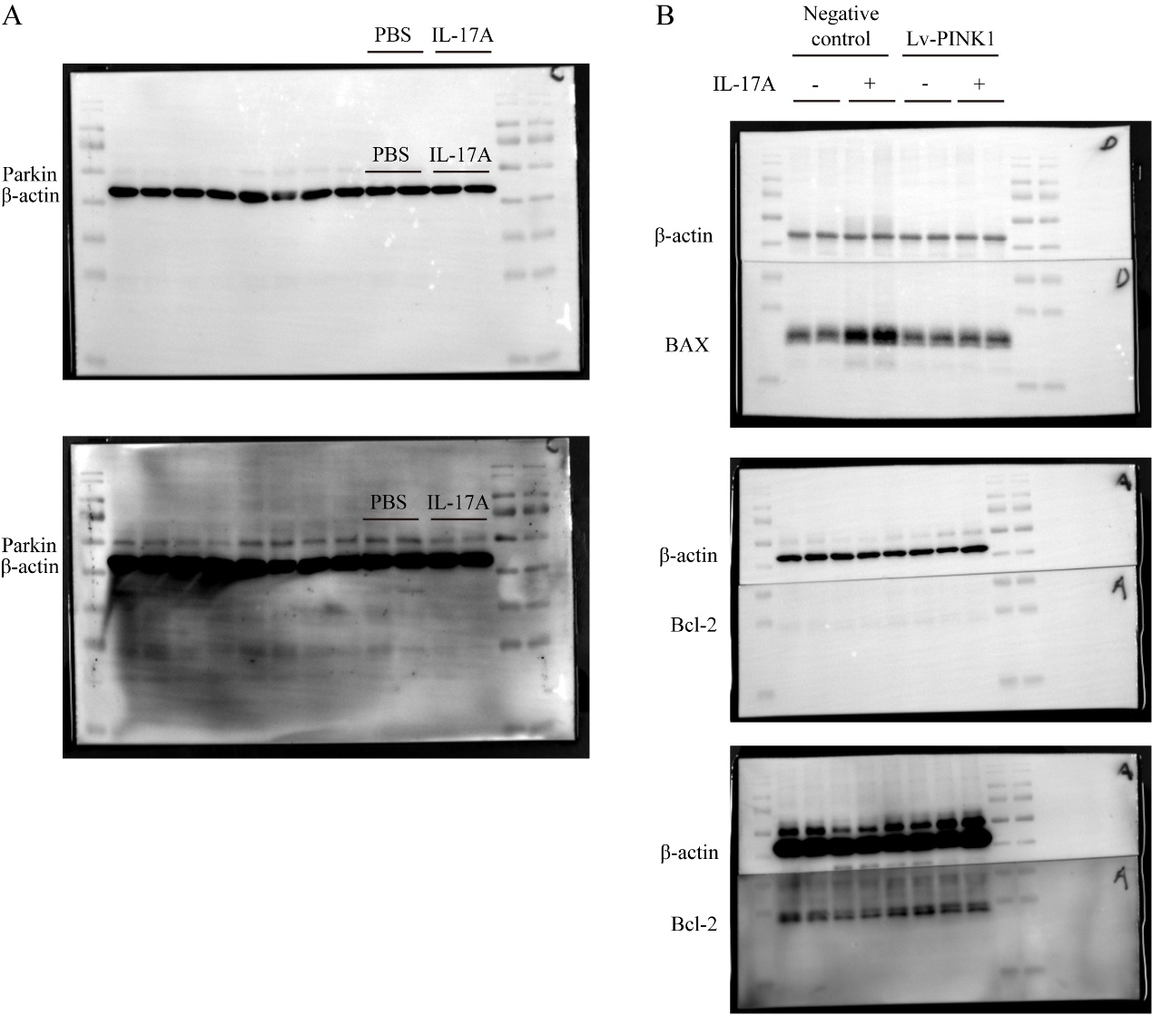


**Supplementary figure 3. Uncropped images of the immunoblot gels for figure 5.**
